# Supplementary material for: Psychological Impact of TP53-Variant-Carrier Newborns and Counselling on Mothers: A Pediatric Surveillance Cohort
Source: Cancers (Basel). 2022 Jun 15;14(12):2945. doi: 10.3390/cancers14122945 (PMC9221115; doi:10.3390/cancers14122945)
Supplement: Supplementary file 1 [file cancers-14-02945-s001.zip › cancers-1724527-supplementary.pdf]

Article

Psychological impact of *TP53* variant-carrier newborns and counselling on mothers: A pediatric surveillance cohort

Supplementary Material

Genetic testing and counselling focused on the germline *TP53* p.R337H variant

In a revision of the genetic testing guidelines, the American Society of Clinical Oncology (ASCO) reiterates the need to differentiate between routine and cancer risk-associated tests [1]. These authors underlined eight important recommendations (Supplementary Table S1) that guarantee safer and clinically appropriate results. Our genetic testing protocol followed these criteria and adapted to the counselling method. These features include: the *TP53* p.R337H frequency among all pediatric adrenocortical carcinoma (ACT) patients is about 90–95% [2,3] and the high frequency of this mutation (0.27–0.30%) among all tested newborns in the state of Paraná [4,5]; the tests (PCR-RFLP or Real-time PCR) are accurately interpreted and confirmed using DNA sequencing. The test results are of clinical utility and we focus our surveillance on ACT and other pediatric cancers. A simplified protocol without periodical exams was presented to the parents [5,6], with increased survival rates due to early stage pediatric ACT diagnosis.

Carriers of the *TP53* p.R337H allele have an increased risk of developing pediatric ACT, and the risk drops dramatically to the other most common pediatric malignancies, choroid plexus carcinoma (65% of all cases), neuroblastoma (8.4% of all cases) and osteosarcoma (7.3% of all cases) [7-9]. The tumor types are more common in adults, including breast carcinoma, gastric, prostate, lung, sarcomas, brain tumors, colon, and oesophagus cancers [4,10-15]. Family history of cancer associated with the p.R337H allele range from no cancer or isolated cancer cases to those fulfilling classic criteria for Li-Fraumeni syndrome. The strong susceptibility of the immature adrenal cortex to develop ACT in p.R337H carriers in a short period of 2-5 years contrasts with ACT lower frequency in adults [16]. Counselling with an intense education and communication programme with the parents without periodical exams was successful to diagnose early onset ACT in children of the surveillance program [5,6]. PCR-RFLP is easily interpreted, affordable, highly sensitive, almost always confirmed by DNA sequencing, and of clinical utility to evaluate tumor penetrance and increase the cure rate [8].
The main objective of the counselling protocol is to educate and assist p.R337H-positive carriers, showing that in some individuals or families, the risk is sometimes enriched by other variants and environmental factors. This process is more effective when the pedigrees are already fully informative, covering at least four generations, usually taking a few months and several contacts with the families to be completed. Oncologists, physicians, geneticists, nurses, psychologists, and social workers have strived for more than 18 years to find out that associated variants may have contributed to modifying p.R337H penetrance. Counselled carriers attended educational processes exploring a face-to-face dialogue, aimed at reducing the psychological impact in relation to the DNA test result and/or report of cancer diagnosis. The participant was invited for other DNA tests when the cancer profile (e.g., multiple cancer types) is compatible with a classic LFS. As these associations are still hypothetical, they are treated superficially during these sessions until proven. We maintained contact with all relatives, and mainly the parents of children <5 years of age. Only carriers presenting with signs and symptoms were invited to clinical, hormonal, and imaging exams [5,6]. Thus, counselling attempts to distinguish and explain cancer risks in p.R337H carriers in over 600 germline p.R337H families, or based on possible associations with other genetic variants or environmental pollutants.

Post the pre-counselling session (prior to the Guthrie test), mothers were invited to the outpatient clinic for result disclosure, the first genetic counselling session, a second p.R337H test (DNA sequencing) in her newborn together with the first test in the parents. The main reason for this is the higher probability of adrenal cortex tumor development in the first post-natal years, and second, the 30 to 40 times lower chance of plexus choroid carcinoma, neuroblastoma or sarcoma development [7-9]. According to the International Pediatric ACT Registry, most cases are diagnosed before completing 5 years, which included delayed diagnosis for most cases [17]. Post the pre-counselling session (48 h after birth), the initial counselling session included only the parents of the proband (children testing positive for p.R337H), and later extends more comprehensively in clinical details, according to the cultural and educational levels of the parents to the remaining family members on the parental side segregating the p.R337H variant. After the initial session with the parents of the newborn (see T1 in Figure 1), they learned about the signs and symptoms of pediatric or adult ACT patients, as well as signs and symptoms of other malignancies. Some cooperative risk variants have been found, and some were discussed with families with a higher number of cancers later when the pedigree is clearly informative (usually 6 months later). In about 50% of the cases, the parents generally predict which parental side carries the *TP53* p.R337H variant in session 1 (T1) before disclosing the DNA test; however, not rarely, tumor cases predominate in the parental side not segregating R3337H. In the latter case, we explored the environmental role, including the contribution of viruses and bacteria in carcinogenesis (e.g., papilloma virus, Hepatitis B/C virus, Helicobacter pylori).

Education and decision to participate

The need for one or more sessions of genetic counselling would depend on the level of education of the families. The opportunity to reflect and decide later (e.g., whether or not to test the variant later after more comprehensive explanations) generally allowed the participants to discuss the subject with their family members. The decision to participate is strongly influenced by the number of people in the family who have already developed cancer and are *TP53* p.R337H carriers. Contrarily, families with several p.R337H carriers and no cancer occurrence, most often discourage non-participation in genetic counselling. Invited individuals systematically check information to reach a decision on whether to test for the p.R337H variant and whether or not to participate. After presenting several examples of pedigrees, with different types of cancer, in sex- and age-specific groups, we use the closest pedigrees type to predict similarities to the new consulting family. The probability of occurrence of the same type of cancer is high and is more evident for breast and pediatric ACT, the two most frequent cancers [4,10]. The fact that the *TP53* p.R337H variant has a low penetrance allows for less urgent decision time than in a family with classical Li-Fraumeni syndrome (mutations with a higher probability of cancer in patients under 45 and 5 years of age), generally without sparing mutation-bearing generations. It is common for p.R337H-carriers over 60 years old to remain healthy or to fall sick and die from other diseases, which is not usually the case for the classic Li-Fraumeni carrier [18,19].

Participants and topics

The probands are newborns who underwent the Guthrie test after pre-counselling and the mother's approval by signing a consent form. The decision to split counselling over several sessions or a single counselling session is relevant to the participant. The average duration of the sessions was variable according to the willingness, interest, and level of education of the participant. The points explored in counselling topics were partially adapted from Brain et al. (2005) [20].

Counselling topics

(1) Risks of cancer in children and adults based on evidence: breast, stomach, ACT, intestine, brain, oesophagus, prostate, lung, sarcomas, liver, uterus, ovaries, among other less common cancers. The database used to illustrate each cancer profile per pedigree includes p.R337H-carriers from 0 to 91 years of age, and more than 50% who never had cancer. Therefore, it is explained that the probability

of not having a cancer is approximately similar to the probability of having the disease before 60 years of age. This information is repeated a few times, which is a relief for the participants to know that they have a less dramatic outcome than the stigmatised classical LFS. These findings are in line with the consensus described as heritable *TP53*-related cancer syndrome [21]. We discuss the predominant age groups, gender differences, and signs and symptoms for each type of cancer. These topics would last more than 20 min, according to the questions or need to clarify doubts. It is explained that each family has its own profile of tendencies, ranging between families with many cancer cases and families without cancer. Explanations, guidelines, and recommendations for preventing other risks are based on gender, age range, life habits, degree of environmental exposure, chronic infections (*Helicobacter pylori,* Epstein-Barr, hepatitis B virus, hepatitis C virus, human papilloma virus, smoking), and the need to investigate possible associations with other genetic variants. Among the new variants, a new founder, *XAF1*-E134*, co-segregates with *TP53* p.R337H in more than 70% of the carriers, may increase risk to sarcoma, but in much lower risk to ACT [22].
(2) Risk of cancer in the general population supposedly without the p.R337H variant (further stressing the role of environmental factors and other genetic and epigenetic changes). Here we conclude that many of our families with the *TP53* p.R337H variant may have fewer cases of cancer than in many communities supposedly exposed only to environmental factors.
(3) *TP53* p.R337H Gene Testing Options: Two drops of blood were obtained from the child's foot (Guthrie test) up to three (3) years of age and, after this age, the finger was punctured. When the test was positive for p.R337H, it was explained that the test result was confirmed in conjunction with that for *XAF1*-E134*, because it is located close to the *TP53* p.R337H haplotype and both co-segregate in ~70–100% of the tested patients [22].
(4) Surveillance after a positive test result. It is sought to demonstrate that each type of cancer has a most common onset time, and that the adult participant does not need to be followed up on an outpatient basis, but could contribute by passing information to a physician about signs that suggest suspicion of cancer. In the first part of counselling, they learn about the signs and symptoms suggestive of cancer (and this dialogue is repeated in the second session).
(5) The genetic implications for family members, considering age and gender, here we explain the probabilities of the individual carrying the p.R337H variant and what it represents (item 1 above).
(6) Discussion about test rejection: It is justified that this decision has already been made by other people of the same family (examples are given to other families), and that the participant's decision will be respected. It is sought to avoid psychological reactions, either in rejection of the test, or to those who had to test the mutation and exhibited a positive result for the mutation. We present several projections on how to cope with positivity of the mutation and the rejection (by our group or with other doctors).
(7) Discussion in favour of performing DNA tests: The advantages are presented and discussed.
(8) Scenarios of families already treated (without identification) with or without cancer, are illustrated in pedigrees. The objective is to show that the penetrance of the p.R337H variant is low. In general, we present the number of people carrying the p.R337H variant in large, medium, and small families, who had cancer detected at early or advanced stages, and their outcomes.
(9) Individualisation of care: The purpose is to guide and recommend possible examinations according to the profile of each family member. Adult patients are referred to other hospitals. Pediatric patients are managed at the Pequeno Príncipe Hospital or Erasto Gartner Hospital (Curitiba, Brazil) or according to the family's preferences and the availability of specialists in their place of residence.
(10) Optional assessment and management of depression and/or anxiety in mothers of children positive for p.R337H.

In general, acceptance and level of satisfaction of the participants was good. In addressing emotional issues, counsellors tend to provide participants with possible topics for introspection within an informational framework. Participants were asked to engage in positive reflections and practical actions.

REFERENCES

1. Robson, M.E.; Storm, C.D.; Weitzel, J.; Wollins, D.S.; Offit, K.; American Society of Clinical Oncology. American Society of Clinical Oncology Policy statement update: genetic and genomic testing for cancer susceptibility. *J Clin Oncol* **2010,** *28*, 893. doi: 10.1200/JCO.2009.27.0660.
2. Ribeiro, R.C.; Sandrini, F.; Figueiredo, B.; Zambetti, G.P.; Michalkiewicz, E.; Lafferty, A.R.; DeLacerda, L.; Rabin, M.; Cadwell, C.; Sampaio, G.; Cat, I.; Stratakis, C.A.; Sandrini, R. An inherited p53 mutation that contributes in a tissue-specific manner to pediatric adrenal cortical carcinoma. *Proc Natl Acad Sci U S A* **2001,** *98,* 9330. doi: 10.1073/pnas.161479898.
3. Pereira, R.M.; Michalkiewicz, E.; Sandrini, F.; Figueiredo, B.C.; Pianovski, M.; França, S.N.; Boguszewski, M.C.S.; Costa, O.; Cat, I.; de Lacerda Filho, L.; Sandrini, R. Childhood adrenocortical tumors. *Arq Bras Endocrinol Metabol* **2004,** *48*, 651. doi: 10.1590/s0004-27302004000500010.
4. Custódio, G.; Parise, G.A.; Kiesel Filho, N.; Komechen, H.; Sabbaga, C.C.; Rosati, R.; Grisa, L.; Parise, I.Z.S.; Pianovski, M.A.D.; Fiori, C.M.C.M.; Ledesma, J.A.; Barbosa, J.R.S.; Figueiredo, F.R.O.; Sade, E.R.; Ibañez, H.; Arram, S.B.I.; Stinghen, S.T.; Mengarelli, L.R.; Figueiredo, M.M.O.; Carvalho, D.C.; Avilla, S.G.A.; Woiski, T.D.; Poncio, L.C.; Lima, G.F.R; Pontarolo, R.; Lalli, E.; Zhou, Y.; Zambetti, G.P.; Ribeiro, R.C.; Figueiredo, B.C. Impact of neonatal screening and surveillance for the TP53 R337H mutation on early detection of childhood adrenocortical tumors. *J Clin Oncol* **2013,** *31*, 2619. doi: 10.1200/JCO.2012.46.3711.
5. Costa, T.E.J.; Gerber, V.K.Q.; Ibañez, H.C.; Melanda, V.S.; Parise, I.Z.S.; Watanabe, F.M.; Pianovski, M.A.D.; Fiori, C.M.C.M.; Fabro, A.L.M.R.; da Silva, D.B.; Andrade, D.P.; Komechen, H.; Mendes, M.C.; Carboni, E.; Kuczynski, A.P.; Souza, E.N.; Paraizo, M.M.; Ibañez, M.V.C.; Castilho, L.M.; Cruz, A.F.; da Maia, T.F.; Machado-Souza, C.; Rosati, R.; Oliveira, C.S.; Parise, G.A.; Passos, J.D.C.; Barbosa, J.R.S.; Figueiredo, M.M.O.; Lima, L.; Tormen, T.; Sabbaga, C.C.; Ávilla, S.G.A.; Grisa, L.; Aranha, A.; Tosin, K.C.F.; Ogradowski, K.R.P.; Lima, G.; Legal, E.F.; Anegawa, T.H.; Mazzuco, T.L.; Grion, A.L.; Balbinotti, J.H.G.; Dammski, K.L.; Melo, R.G.; Filho, N.K.; Custódio, G.; Figueiredo, B.C. Penetrance of the *TP53* R337H mutation and pediatric adrenocortical carcinoma incidence associated with environmental influences in a 12-year observational cohort in Southern Brazil. *Cancers* **2019,** *11*, 1894. doi: 10.3390/cancers11111804.
6. Tosin, K.C.F.; Legal, E.F.; Pianovski, M.A.D.; Ibañez, H.C.; Custódio, G.; Carvalho, D.S.; Figueiredo, M.M.O.; Filho, A.H.; Fiori, C.M.C.M.; Rodrigues, A.L.M.; Mello, R.G.; Ogradowski, K.R.P.; Parise, I.Z.S.; Costa, T.E.J.; Melanda, V.S.; Watanabe, F.M.; Silva, D.B.; Komechen, H.; Laureano, H.A.; Carboni, E.K.; Kuczynski, A.P.; Luiz, G.C.F.; Lima, L.; Tormen, T.; Gerber, V.K.Q.; Anegawa, T.H.; Avilla, S.G.A.; Tenório, R.B.; Mendes, E.L.; Donin, R.D.F.; Souza, J.; Kozak,V.N.; Oliveira, G.S.; Souza, D.C.; Gomy, I.; Teixeira, V.B.; Borba, H.H.L.; Filho, N.K.; Parise, G.A.; Ribeiro, R.C.; Figueiredo, B.C. Newborn screening for the detection of the TP53 R337H variant and surveillance for early diagnosis of pediatric adrenocortical tumors: Lessons learned and way forward. *Cancers* **2021,** *13*, 6111. doi: 10.3390/cancers13236111.
7. Seidinger, A.L.; Mastellaro, M.J.; Paschoal Fortes, F.; Assumpção, J.D.; Cardinalli, I.A.; Ganazza, M.A.; Ribeiro, R.C.; Brandalise, S.R.; dos Santos Aguiar, A.; Yunes, J.A. Association of the highly prevalent TP53 R337H mutation with pediatric choroid plexus carcinoma and osteosarcoma in southeast Brazil. *Cancer* **2011,** *117*, 2228. doi: 10.1002/cncr.25826.
8. Custódio, G.; Taques, G.R.; Figueiredo, B.C.; Gugelmin, E.S.; Figueiredo, M.M.O.; Watanabe, F.; Pontarolo, R.; Lalli, E.; Torres, L.F.B. Increased incidence of choroid plexus carcinoma due to the germline TP53 R337H mutation in southern Brazil. *PLoS ONE* **2011,** *6,* e18015. doi: 10.1371/journal.pone.0018015.

9. Seidinger, A.L.; Fortes, F.P.; Mastellaro, M.J.; Cardinalli, I.A.; Zambaldi, L.G.; Aguiar, S.S.; Yunes, J.A. Occurrence of Neuroblastoma among TP53 p.R337H Carriers. *PLoS One* **2015,** *10*, e0140356. doi: 10.1371/journal.pone.0140356.
10. Figueiredo, B.C.; Sandrini, R.; Zambetti, G.P.; Pereira, R.M.; Cheng, C.; Liu, W.; Lacerda, L.; Pianovski, M.A.; Michalkiewicz, E.; Jenkins, J.; Rodriguez-Galindo, C.; Mastellaro, M.J.; Vianna, S.; Watanabe, F.; Sandrini, F.; Arram, S.B.I.; Boffetta, P.; Ribeiro, R.C. Penetrance of adrenocortical tumours associated with the germline TP53 R337H mutation. *J Med Genet* **2006,** *43*, 91. doi: 10.1136/jmg.2004.030551.
11. Palmero, E.I.; Schüler-Faccini, L.; Caleffi, M.; Achatz, M.I.W.; Olivier, M.; Martel-Planche, G.; Marcel, V.; Aguiar, E.; Giacomazzi, J.; Ewald, I.P.; Giugliani, R.; Hainaut, P.; Ashton-Prolla, P. Detection of R337H, a germline TP53 mutation predisposing to multiple cancers, in asymptomatic women participating in a breast cancer screening program in Southern Brazil. *Cancer Lett* **2008,** *261*, 21. doi: 10.1016/j.canlet.2007.10.044.
12. Gomes, M.C.; Kotsopoulos, J.; de Almeida, G.L.; Costa, M.M.; Vieira, R.; de Ag Filho, F.; Pitombo, M.B.; Leal, P.R.F.; Royer, R.; Zhang, P.; Narod, S.A. The R337H mutation in TP53 and breast cancer in Brazil. *Hered Cancer Clin Pract* **2012,** *10*, 3. doi: 10.1186/1897-4287-10-3.
13. Cury, N.M.; Ferraz, V.E.; Silva, W.A. Jr. TP53 p.R337H prevalence in a series of Brazilian hereditary breast cancer families. *Hered Cancer Clin Pract* **2014,** *12*, 8. doi: 10.1186/1897-4287-12-8.
14. Couto, P.P.; Bastos-Rodrigues, L.; Schayek, H.; Melo, F.M.; Lisboa, R.G.C.; Miranda, D.M.; Vilhena, A.; Bale, A.E.; Friedman, E.; De Marco, L. Spectrum of germline mutations in smokers and non-smokers in Brazilian non-small-cell lung cancer (NSCLC) patients. *Carcinogenesis* **2017,** *38*, 1112. doi: 10.1093/carcin/bgx089.
15. Mastellaro, M.J.; Seidinger, A.L.; Kang, G.; Abrahão, R.; Miranda, E.C.M.; Pounds, S.B.; Cardinalli, I.A.; Aguiar, S.S.; Figueiredo, B.C.; Rodriguez-Galindo, C.; Brandalise, S.R.; Yunes, J.A.; de A Barros-Filho, A.; Ribeiro, R.C. Contribution of the TP53 R337H mutation to the cancer burden in southern Brazil: insights from the study of 55 families of children with adrenocortical tumors. *Cancer* **2017,** *123*, 3150–3158. doi: 10.1002/cncr.30703.
16. Latronico, A.C.; Pinto, E.M.; Domenice, S.; Fragoso, M.C.; Martin, R.M.; Zerbini, M.C.; Lucon, A.M.; Mendonca, B.B. An inherited mutation outside the highly conserved DNA-binding domain of the p53 tumor suppressor protein in children and adults with sporadic adrenocortical tumors. *J Clin Endocrinol Metab* **2001,** *86*, 4970–4973. doi: 10.1210/jcem.86.10.7957.
17. Michalkiewicz, E.; Sandrini, R.; Figueiredo, B.; Miranda, E.C.M.; Caran, E.; Oliveira-Filho, A.G.; Marques, R.; Pianovski, M.A.D.; Lacerda, L.; Cristofani, L.M.; Jenkins, J.; Rodriguez-Galindo, C.; Ribeiro, R.C. Clinical and outcome characteristics of children with adrenocortical tumors: a report from the International Pediatric Adrenocortical Tumor Registry. *J Clin Oncol* **2004,** *22*, 838-845. doi: 10.1200/JCO.2004.08.085.
18. Wu, C.C.; Shete, S.; Amos, C.I.; Strong, L.C. Joint effects of germ-line p53 mutation and sex on cancer risk in Li–Fraumeni syndrome. *Cancer Res* **2006,** *66*, 8287. doi: 10.1158/0008-5472.CAN-05-4247.
19. Bougeard, G.; Renaux-Petel, M.; Flaman, J.M.; Charbonnier, C.; Fermey, P.; Belotti, M.; Gauthier-Villars, M.; Stoppa-Lyonnet, D.; Consolino, E.; Brugières, L.; Caron, O.; Benusiglio, P.R.; Bressac-de Paillerets, B.; Bonadona, V.; Bonaïti-Pellié, C.; Tinat, J.; Baert-Desurmont, S.; Frebourg, T. Revisiting Li–Fraumeni syndrome from TP53 mutation carriers. *Clin Oncol* **2015,** *33*, 2345. doi: 10.1200/JCO.2014.59.5728.
20. Brain, K.; Sivell, S.; Bennert, K.; Howell, L.; Howell, L.; France, L.; Jordan, S.; Rogers, M.T.; Gray, J.; Sampson, J. An exploratory comparison of genetic counselling protocols for HNPCC predictive testing. *Clin Genet* **2005,** *68*, 255. doi: 10.1111/j.1399-0004.2005.00491.x.

21. Frebourg, T.; Bajalica Lagercrantz, S.; Oliveira, C.; Magenheim, R.; Evans, D.G.; European Reference Network GENTURIS. European Reference Network GENTURIS (2020). Guidelines

for the Li–Fraumeni and heritable TP53-related cancer syndromes. *Eur J Hum Genet* **2020,** *28,* 1379. doi: 10.1038/s41431-020-0638-4.

22. Pinto, E.M.; Figueiredo, B.C.; Chen, W.; Galvao, H.C.R.; Formiga, M.N.; Fragoso, M.C.B.V.; Ashton-Prolla, P.; Ribeiro, E.M.S.F.; Felix, G.; Costa, T.E.B.; Savage, S.A.; Yeager, M.; Palmero, E.I.; Volc, S.; Salvador, H.; Fuster-Soler, J.L.; Lavarino, C.; Chantada, G.; Vaur, D.; Odone-Filho, V.; Brugières, L.; Else, T.; Stoffel, E.M.; Maxwell, K.N.; Achatz, M.I.; Kowalski, L.; de Andrade, K.C.; Pappo, A.; Letouze, E.; Latronico, A.C.; Mendonca, B.B.; Almeida, M.Q.; Brondani, V.B.; Bittar, C.M.; Soares, E.W.S.; Mathias, C.; Ramos, C.R.N.; Machado, M.; Zhou, W.; Jones, K.; Vogt, A.; Klincha, P.P.; Santiago, K.M.; Komechen, H.; Paraizo, M.M.; Parise, I.Z.S.; Hamilton, K.V.; Wang, J.; Rampersaud, E.; Clay, M.R.; Murphy, A.J.; Lalli, E.; Nichols, K.E.; Ribeiro, R.C.; Rodriguez-Galindo, C.; Korbonits, M.; Zhang, J.; Thomas, M.G.; Connelly, J.P.; Pruett-Miller, S.; Diekmann, Y.; Neale, G.; Wu, G.; Zambetti, G.P. XAF1 as a modifier of p53 function and cancer susceptibility. *Sci Adv* **2020,** *6,* 3231. doi: 10.1126/sciadv.aba3231.

TABLES

Supplementary Table S1: modified from ASCO Recommendations (Robson et al., 2010)

ASCO Recommendations for genetic and genomic testing on Cancer Risk	
Recommendation 1	The participation of oncologists and other health professionals
Recommendation 2	Promote education and training
Recommendation 3	Conduct research for clinical cases without established criteria
Recommendation 4	Pre- and post-test counselling
Recommendation 5	Use of informed consent
Recommendation 6	Accessibility of the genetic test to the population
Recommendation 7	Confidentiality and privacy
Recommendation 8	Regulation of genetic tests: PCR-RFLP + confirmation of positive results for *TP53* p.R337H using DNA sequencing method (pre- and post-test counselling, respectively)

FIGURES AND FIGURE LEGENDS

Supplementary Figure S1.

Significant difference (Wilcoxon test) was detected in HADS-A between groups of pregnant women according the number of children.

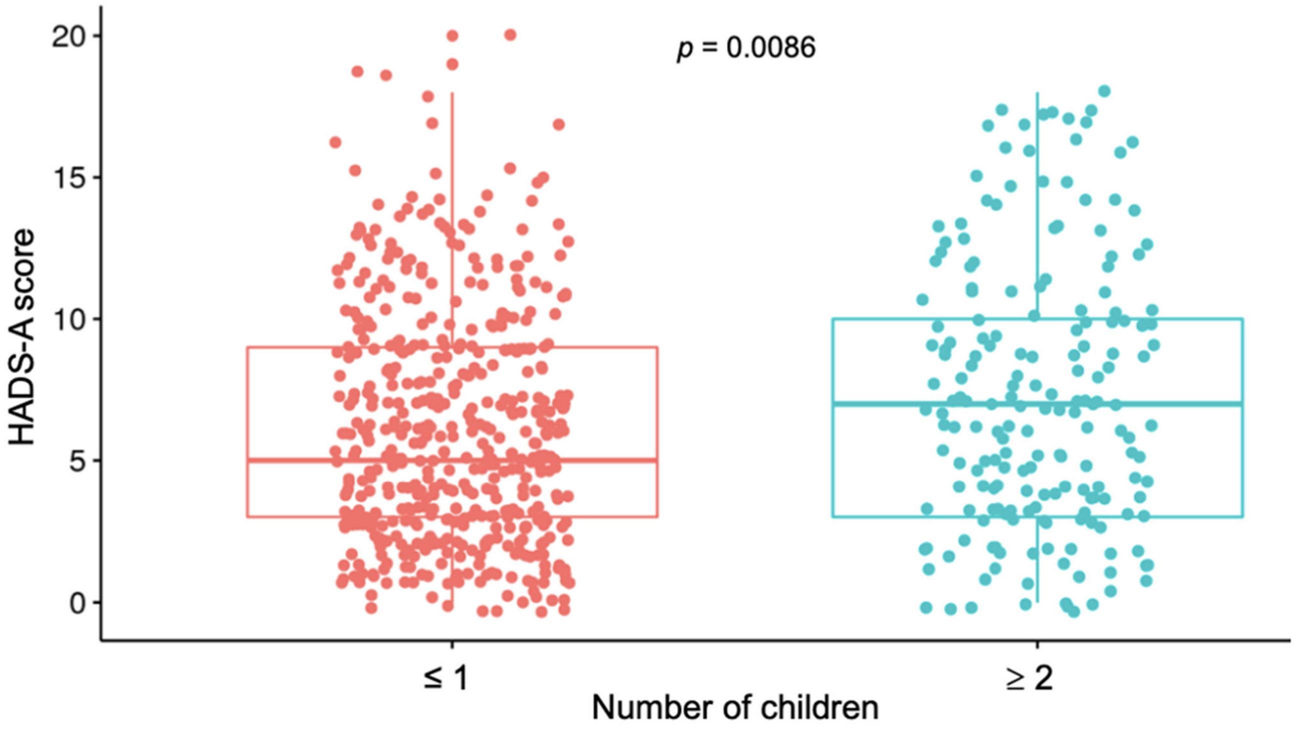

Supplementary Figure S2.
Significant difference (Wilcoxon test) was detected in HADS-D between groups of pregnant women according the number of children.

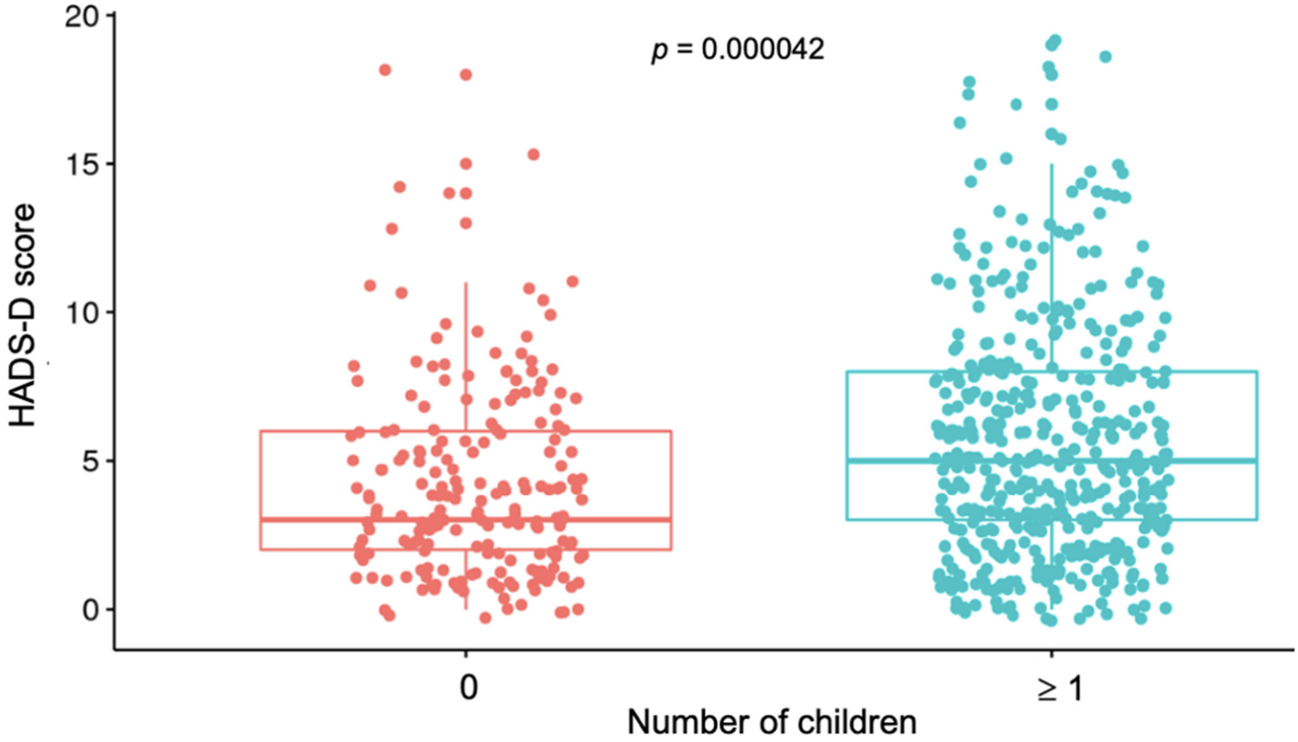

Supplementary Figure S3
Significant difference was detected in HADS-A between groups of pregnant women according the marital status

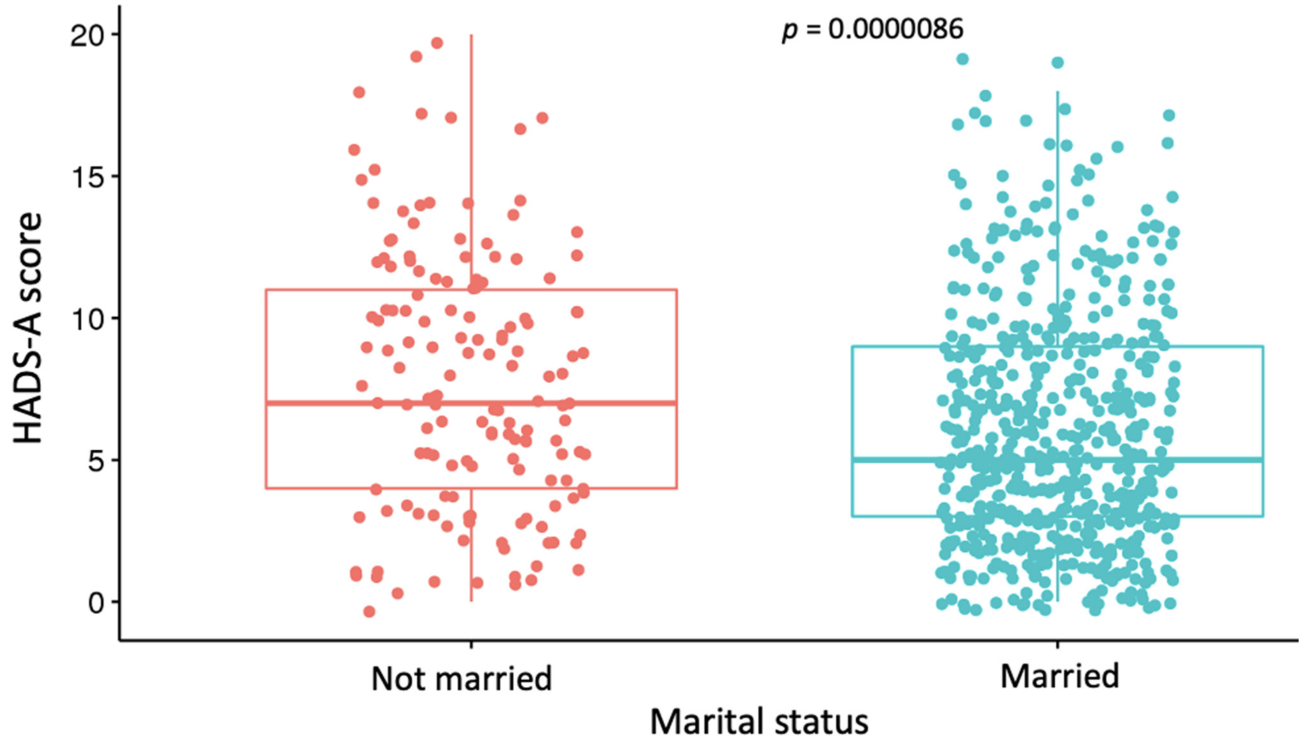

Supplementary Figure S4

Significant difference was detected in HADS-D between groups of pregnant women according the marital status

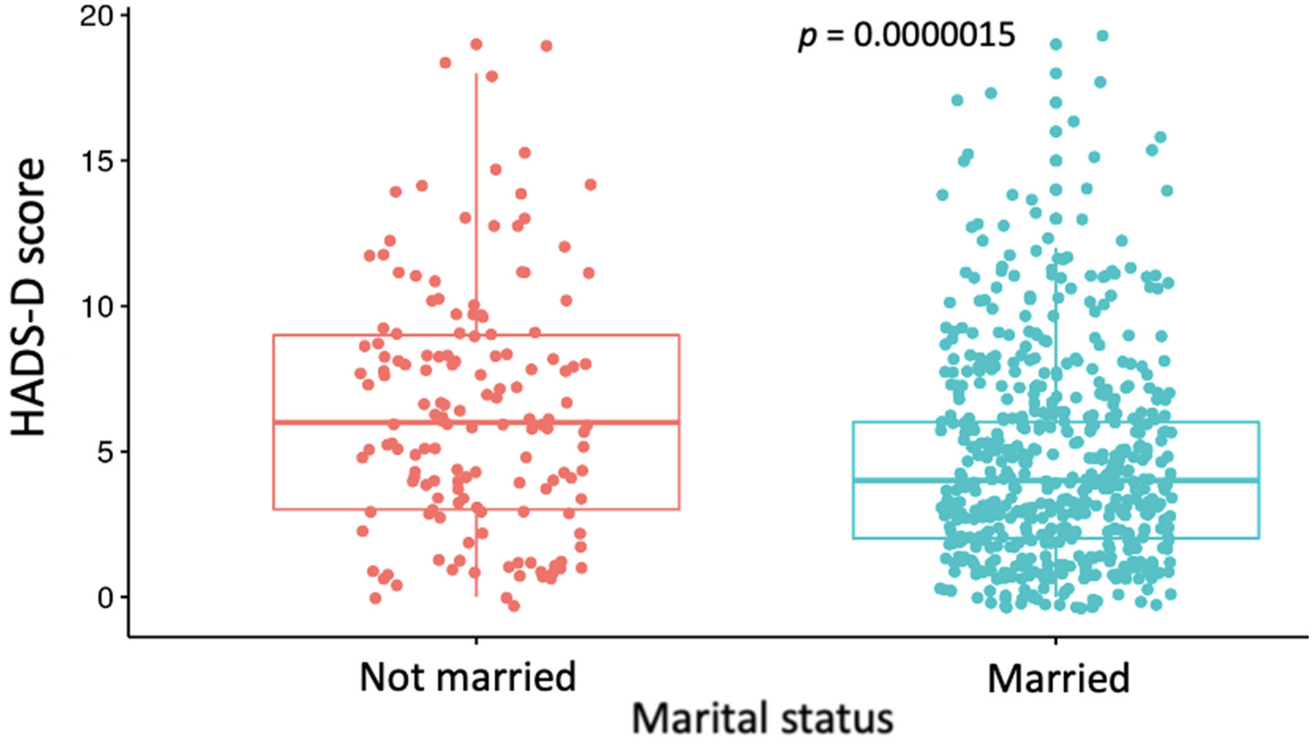

Supplementary Figure S5

Significant difference was detected in HADS-A between groups of pregnant women according the educational level.

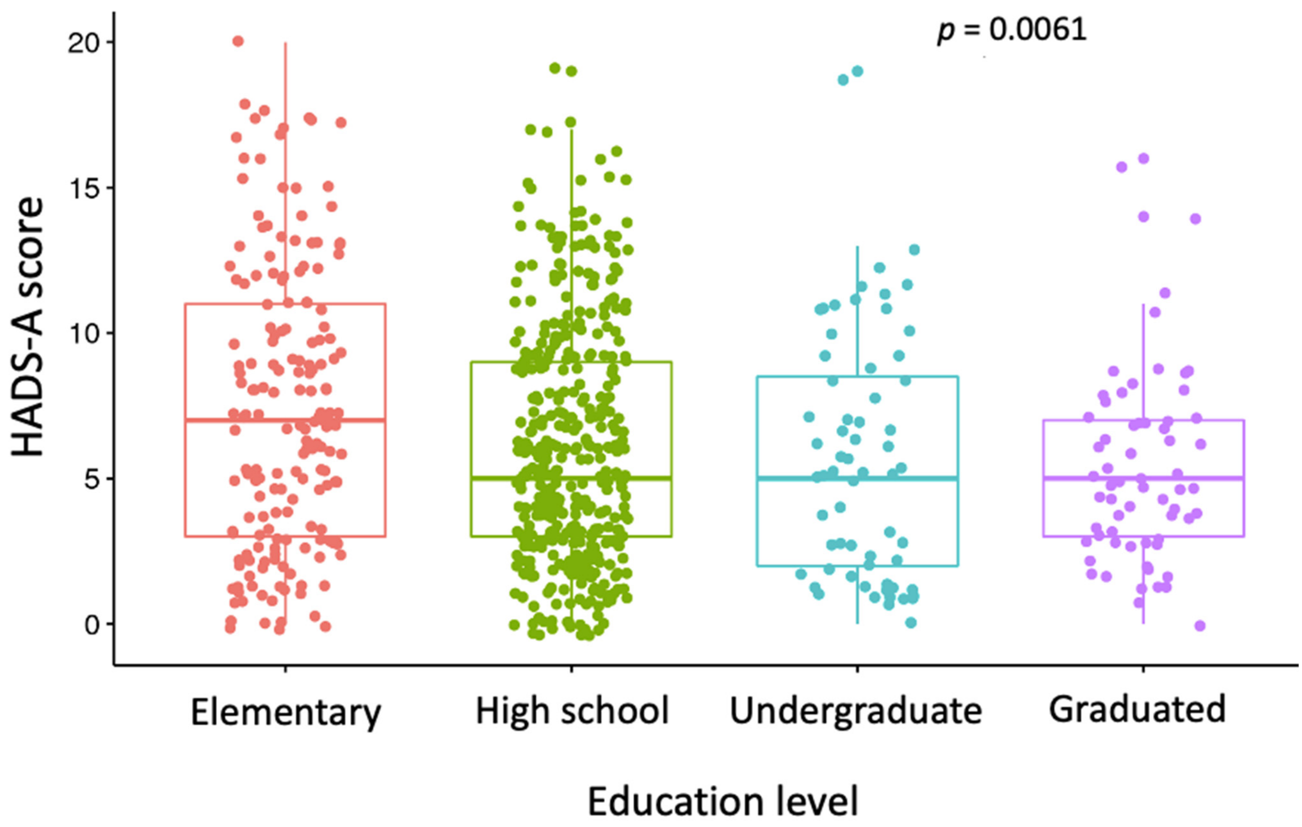

Supplementary Figure S6
Significant difference was detected in HADS-D between groups of pregnant women according the educational level.

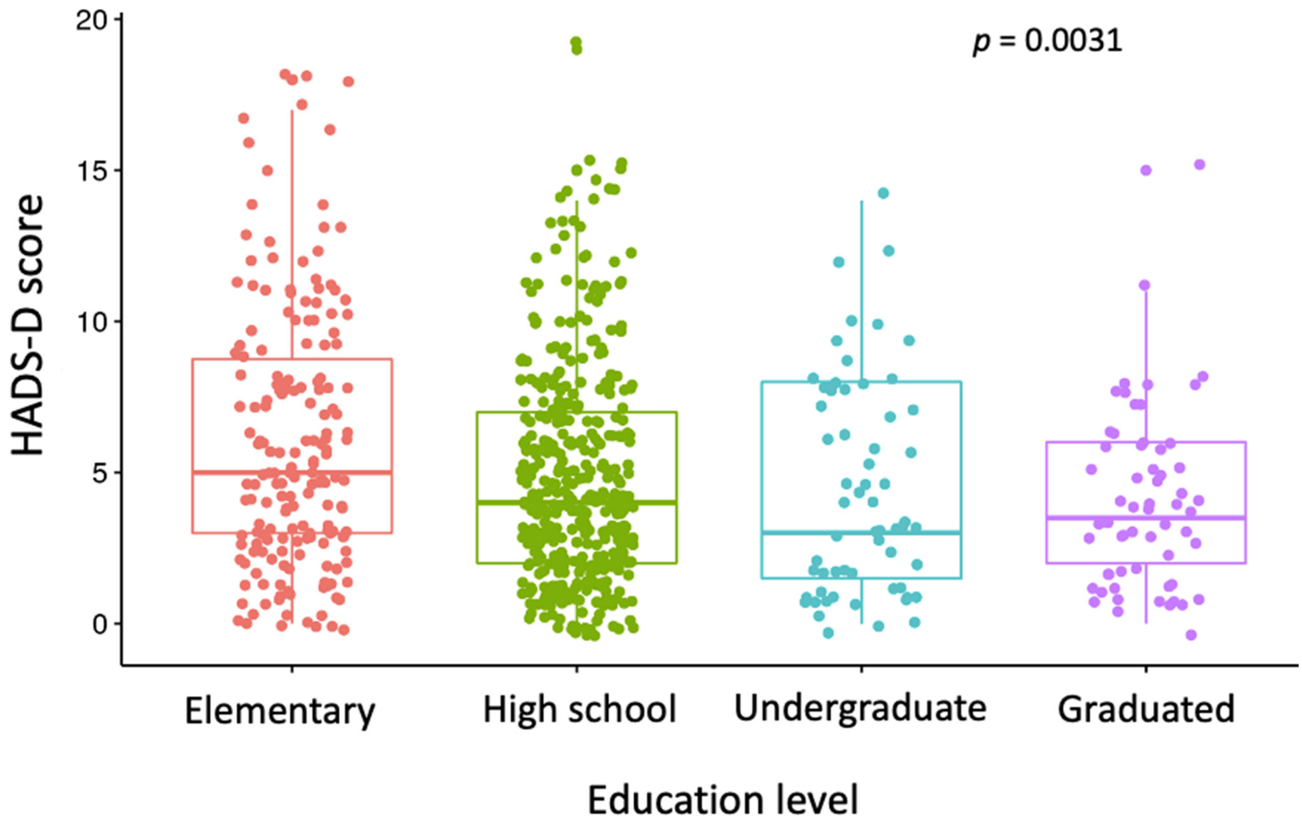

Author Contributions: Conceptualization and formal analysis, A.S.G., C.W.Z., R.G.M., and B.C.F.; funding acquisition, R.G.M. and B.C.F.; investigation, A.S.G., C.W.Z., I.A.M.C., T.C.S., L.R.S.W., K.R.P.O., K.L.D., H.K., M.C.M., E.N.S., M.M.P., I.Z.S.P., G.A.P., A.L.G., G.C., R.G.M., and B.C.F.; methodology, A.S.G., C.W.Z., A.L.G., K.R.P.O., A.L.G., G.C., R.G.M., and B.C.F.; project administration, A.S.G., R.G.M., and B.C.F.; software, A.S.G. and B.C.F.; supervision, A.S.G., R.G.M., and B.C.F.; writing—original draft preparation, A.S.G., C.W.Z., and I.A.M.C.; writing—review and editing, A.S.G., C.W.Z., I.A.M.C., T.C.S., L.R.S., K.R.P.O., H.K., M.C.M., E.N.S., M.M.P., I.Z.S.P., G.A.P., A.L.G., G.C., R.G.M., K.R.P.O., and B.C.F. All authors have read and agreed to the published version of the manuscript.

Funding: This work was funded by Coordenação de Aperfeiçoamento de Pessoal de Nível Superior – Brasil (CAPES) Finance code 001, Conselho National de Desenvolvimento e Pesquisa (CNPq, Brazil), the Ludwig Institute for Cancer Research, and the CNRS EXPOGEN-CANCER. International Associated Laboratory (LIA).

Acknowledgments: The authors are grateful to Ciência Lab, IAP, CEPON, IDAPI and HU-UFSC (Florianópolis, SC, Brazil), for their technical support.

Conflicts of Interest: The authors declare no conflicts of interest.
